# Effect of Particle Size on the Magnetic Properties of Ni Nanoparticles Synthesized with Trioctylphosphine as the Capping Agent

**DOI:** 10.3390/nano6090172

**Published:** 2016-09-13

**Authors:** Toshitaka Ishizaki, Kenichi Yatsugi, Kunio Akedo

**Affiliations:** Toyota Central R&D Labs., Inc., 41-1 Nagakute, Aichi 480-1192, Japan; e1624@mosk.tytlabs.co.jp (K.Y.); akedo@mosk.tytlabs.co.jp (K.A.)

**Keywords:** Ni, nanoparticle, superparamagnetism, phosphine, chemical reduction method, coercive force, saturation magnetization

## Abstract

Magnetic cores of passive components are required to have low hysteresis loss, which is dependent on the coercive force. Since it is well known that the coercive force becomes zero at the superparamagnetic regime below a certain critical size, we attempted to synthesize Ni nanoparticles in a size-controlled fashion and investigated the effect of particle size on the magnetic properties. Ni nanoparticles were synthesized by the reduction of Ni acetylacetonate in oleylamine at 220 °C with trioctylphosphine (TOP) as the capping agent. An increase in the TOP/Ni ratio resulted in the size decrease. We succeeded in synthesizing superparamagnetic Ni nanoparticles with almost zero coercive force at particle size below 20 nm by the TOP/Ni ratio of 0.8. However, the saturation magnetization values became smaller with decrease in the size. The saturation magnetizations of the Ni nanoparticles without capping layers were calculated based on the assumption that the interior atoms of the nanoparticles were magnetic, whereas the surface-oxidized atoms were non-magnetic. The measured and calculated saturation magnetization values decreased in approximately the same fashion as the TOP/Ni ratio increased, indicating that the decrease could be mainly attributed to increases in the amounts of capping layer and oxidized surface atoms.

## 1. Introduction

Magnetic nanoparticles are being studied by a number of researchers owing to their potential applications in a variety of areas including magnetic devices, magnetic fluids, magnetic recording media, medical diagnosis, and hyperthermia [1,2,3,4]. One of the unique characteristics of magnetic nanoparticles is that the coercive force becomes zero at the superparamagnetic regime below a certain critical size because the random flipping of magnetization direction becomes dominant even at room temperature [5,6]. Since hysteresis loss is dependent on the coercive force, a decrease in the size of the magnetic nanoparticles effectively decreases the hysteresis loss [7]. Magnetic materials in the passive components of power control units are necessary to be used under high operating frequencies for next generation power devices in order to improve the performance. Since high operating frequencies enhance hysteresis loss, the use of superparamagnetic nanoparticles in passive components is expected to lead to low loss.

A number of physical and chemical methods are available for producing metal nanoparticles including vapor phase synthesis, mechanical milling, thermal decomposition, electrochemical synthesis, and chemical reduction synthesis. Among the various methods, chemical reduction synthesis is advantageous because the particle size can be effectively controlled in this method by changing the capping and reducing agents and with inexpensive materials and apparatus [8,9]. A number of reports on the chemical reduction synthesis of noble metal nanoparticles have already been published because of the easy reduction of these ions and their high stability in the liquid phase. However, typical magnetic materials, such as Fe, Co, and Ni, are not noble metals. As a result, their metal ions are barely reduced and the particles tend to be unstable. Therefore, the synthesis of magnetic metal nanoparticles with controlled size continues to be challenging. In addition, the dependence of the magnetic properties of these metals on the nanoparticle size needs to be examined.

Among the various magnetic metals, the synthesis of Ni nanoparticles has been considered because Ni ions are reduced relatively easily compared to Fe and Co, and passive layers are generally formed on Ni surfaces that prevent severe oxidation. Strong reducing agents, such as NaBH_4_ and hydrazine, have often been used to synthesize mono-dispersed Ni nanoparticles [10,11,12,13,14,15,16]. However, there are problems related to residual impurities when NaBH_4_ is used and toxicity concerns when hydrazine is used. On the other hand, Ni particles with fewer impurities and less toxicity may be synthesized using polyols. However, in this case, the product particle size tends to be large because the reducing ability of polyols is weak [17,18,19]. Therefore, as an alternate methodology, synthesis using alkylamines has attracted attention because alkylamines not only act as reducing agents and produce products with fewer impurities and less toxicity, but also act as stabilizers for producing nanoparticles [20]. Size-controlled synthesis of Ni nanoparticles can be conducted by changing the amounts of nickel acetylacetonates and alkylamines [21]. In order to decrease the particle size, stabilizers with strong affinity to the Ni surface are generally added. Examples of such stabilizers include phosphines [22,23,24,25,26,27], carboxylic acids [27,28,29,30], and alkylsulfates [30]. Among these functional groups, phosphines are very effective for controlling the particle morphology. However, the effect of Ni nanoparticle size on their magnetic properties has not been clarified adequately so far. Therefore, in the present study, the size-controlled synthesis of Ni nanoparticles using oleylamine (OA) and trioctylphosphine (TOP) was carried out and the effect of particle size on the magnetic properties was examined.

## 2. Results

### 2.1. Synthesis of Ni Nanoparticles

**Figure 1:**
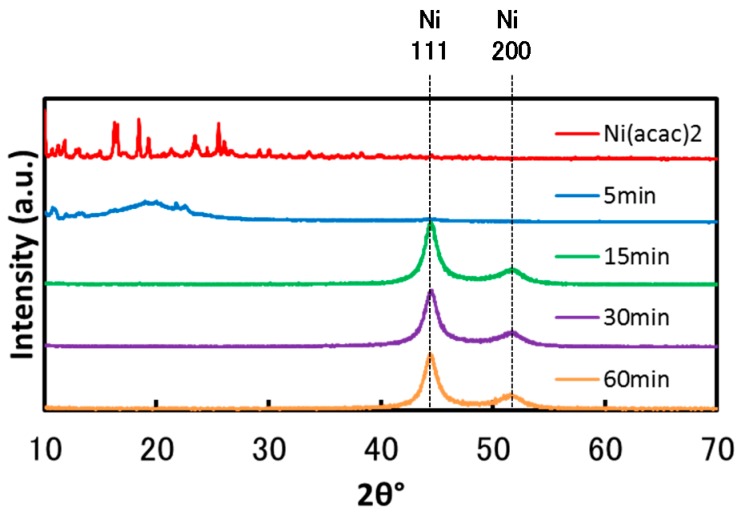
X-ray diffraction (XRD) spectra of the particles obtained at the trioctylphosphine (TOP)/Ni ratio of 0.5 for reaction durations ranging from 5 min to 60 min at 220 °C.

Firstly, the reaction time required to reduce Ni (II) acetylacetonate (Ni(acac)_2_) completely to Ni particles was determined. Figure 1 shows the X-ray diffraction (XRD) spectra of the particles synthesized at the TOP/Ni ratio of 0.5 with the reaction duration ranging from 5 min to 60 min at 220 °C. The XRD pattern of Ni(acac)_2_ was also measured for comparison. For a reaction time of 5 min, there are no peaks corresponding to Ni in the XRD spectrum of the particles. However, unclear peaks are observed in the low angle region. Since the Ni(acac)_2_ spectrum exhibits several peaks in the same region, the particles obtained with a reaction duration of 5 min appear to contain Ni(acac)_2_ and un-known products . Therefore, Ni(acac)_2_ is not completely reduced in 5 min. On the other hand, when the reaction time is over 15 min, the XRD spectra contains peaks corresponding to Ni, whereas there are no peaks related to Ni oxides or Ni(acac)_2_. For a reaction time of 15 min, 2.7 g of particles are obtained, whereas 3.5 g of particles are obtained for reaction durations of 30 min and 60 min. These results suggest that while Ni nanoparticles can be obtained at 15 min, more than 30 min is required to reduce Ni(acac)_2_ completely because 60 mmol of Ni corresponds to 3.5 g. Based on this result, all the subsequent experiments were carried out for a reaction duration of 60 min at 220 °C.

**Figure 2:**
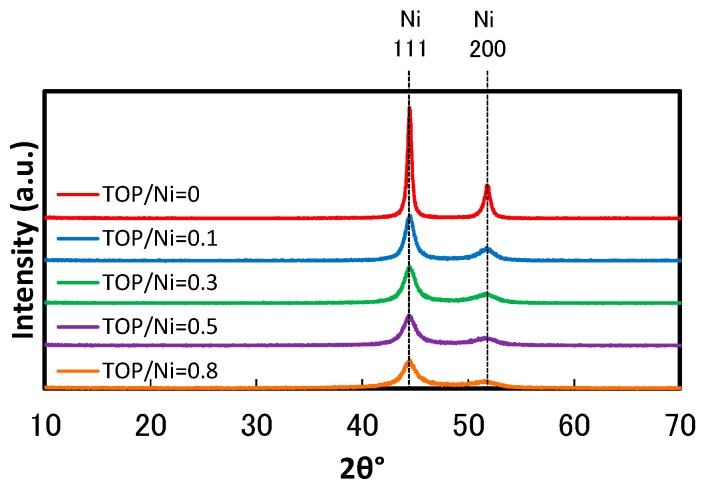
XRD spectra of the Ni nanoparticles at the TOP/Ni ratios in the range of 0–0.8.

**Figure 3:**
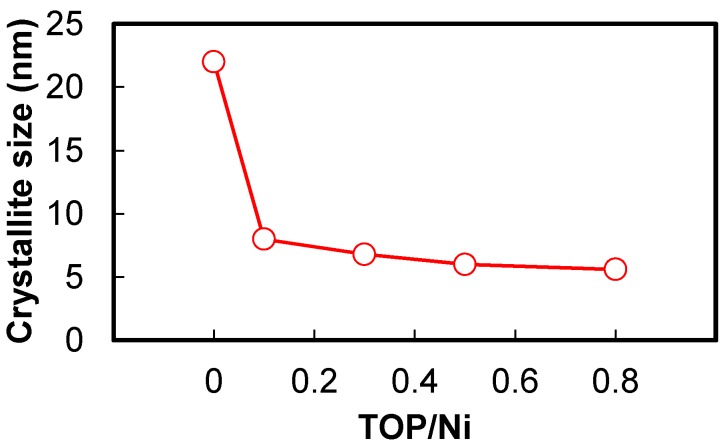
Crystallite sizes of Ni nanoparticles at the TOP/Ni ratios in the range of 0–0.8.

Figure 2 shows the XRD spectra of Ni nanoparticles measured at the TOP/Ni ratios from 0–0.8. All of the XRD spectra exhibit peaks corresponding to Ni and do not contain peaks corresponding to Ni oxide or the raw materials. This suggests that Ni(acac)_2_ is completely reduced in all of the cases. The XRD spectra also suggests that the half bandwidth increases with the increase in the TOP/Ni ratio. Since it is well known that the increase in the half bandwidth corresponds to the decrease in the crystallite size, the XRD spectra indicates that the particles become smaller upon increasing the TOP/Ni ratio. Figure 3 shows the crystallite sizes estimated from the XRD spectra at the TOP/Ni ratios from 0–0.8. This result indicates the declining trend of the crystallite size as the TOP/Ni ratio increases.

**Figure 4:**
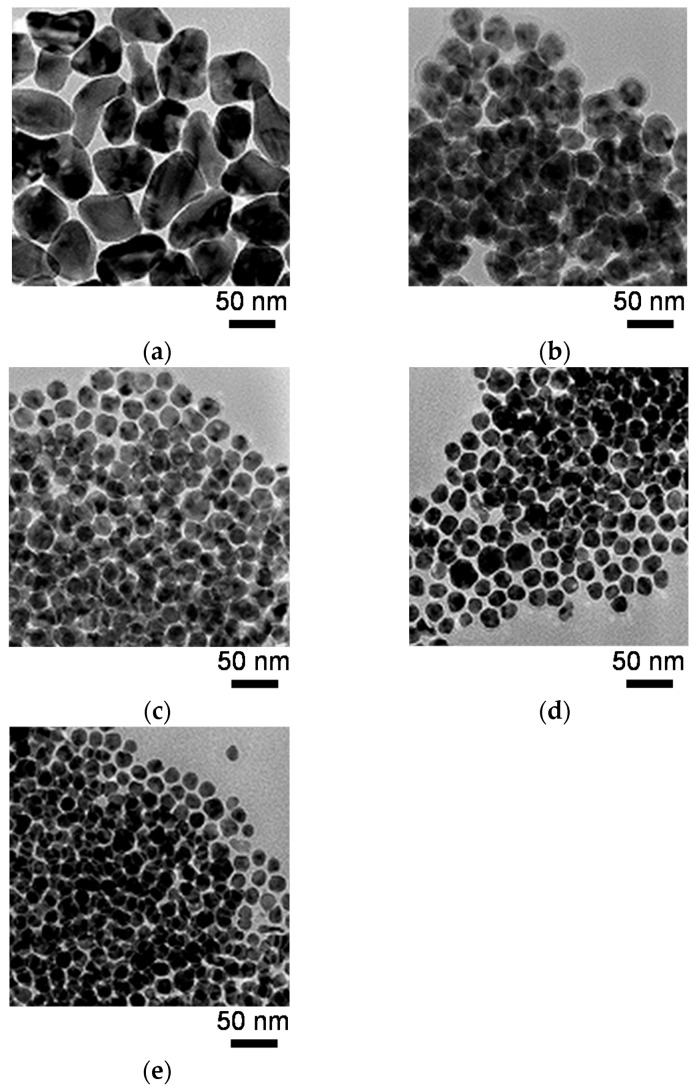
Transmission electron microscopy (TEM) images of the Ni nanoparticles at the TOP/Ni ratios of (**a**) 0; (**b**) 0.1; (**c**) 0.3; (**d**) 0.5; and (**e**) 0.8.

**Figure 5:**
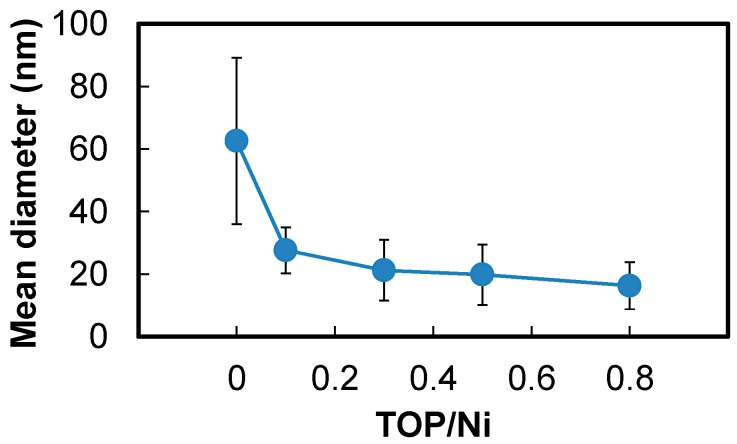
Mean diameters and standard deviations of the particle size of Ni nanoparticles at the TOP/Ni ratios in the range of 0–0.8.

Figure 4 shows transmission electron microscopy (TEM) micrographs of the Ni nanoparticles, whereas Figure 5 shows the mean diameters and standard deviations of particle sizes calculated from the TEM micrographs. The Ni nanoparticle samples used for the TEM studies were fabricated at the TOP/Ni ratios ranging from 0–0.8. The Ni nanoparticles obtained at the TOP/Ni ratio of 0 (i.e., in the absence of TOP) exhibit irregular form, similar to previously reported results for particles synthesized in only hexadecylamine [31]. Since alkylamines readily desorb from the Ni surface owing to weak ligands [25], Ni nanoparticles are prone to agglomeration during reactions at high temperature, as a result of which irregular-shaped particles are obtained. On the other hand, the mean diameters and particle size standard deviations for the Ni nanoparticles drastically decrease upon the addition of TOP. The Ni nanoparticles formed in the presence of TOP are spherical in shape and their size decreases with increase in the TOP/Ni ratio. This trend is quite consistent with the result of the crystallite sizes estimated from the XRD spectra with increase in the TOP/Ni ratio (Figure 3). Therefore, these results indicate that TOP is a very effective additive for controlling the size of the Ni nanoparticles. Since the mean diameter is larger than the crystallite size, overall, it is thought that the obtained Ni nanoparticles are polycrystalline. The mean diameter is particularly larger than the crystallite size at the TOP/Ni ratio of 0. Since OA cannot stabilize Ni surface strongly compared with TOP, we think that the Ni nanoparticles have grown up with agglomeration of initial nuclei remarkably in the absence of TOP so that the particle shape became irregular and the particle size standard deviation was much larger than those with the addition of TOP.

### 2.2. Magnetic Properties of Ni Nanoparticles

**Figure 6:**
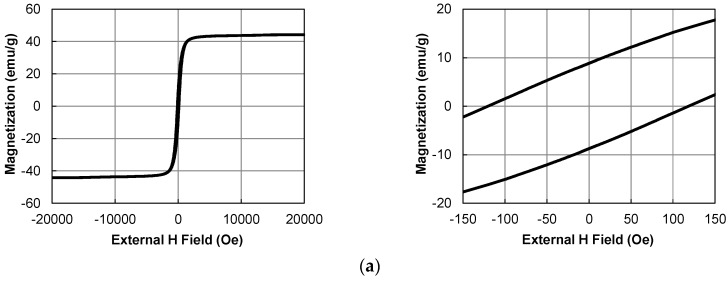

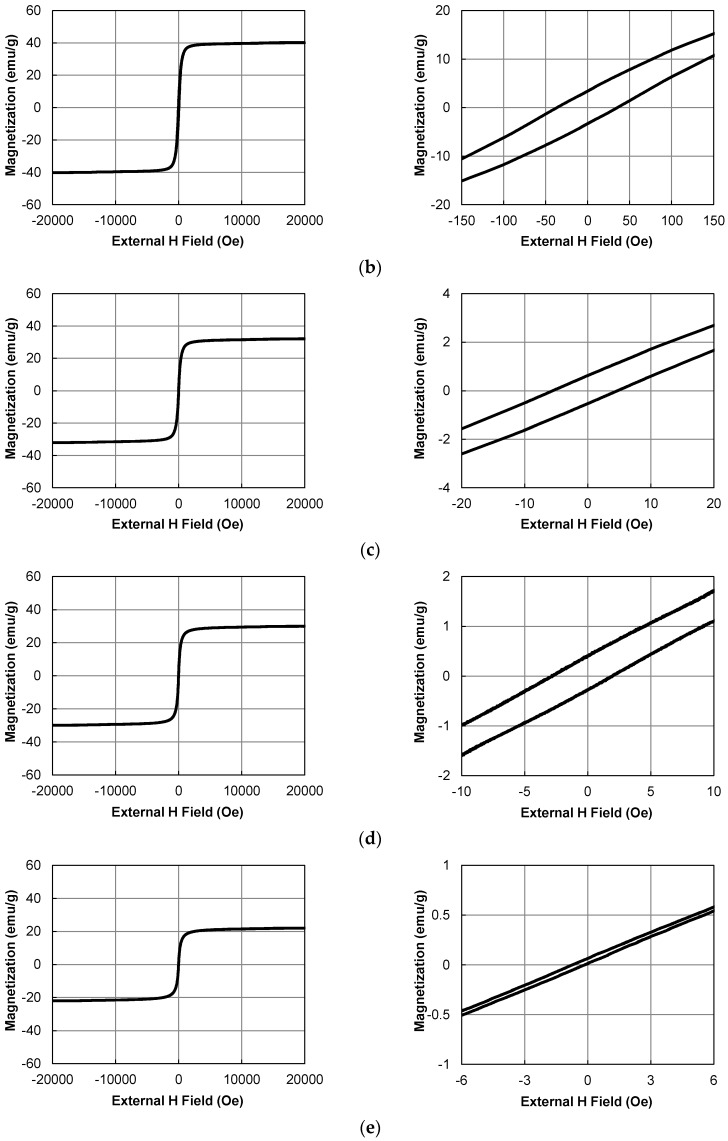
Magnetization curves of the Ni nanoparticles, entire (**left**) and enlarged views (**right**), at the TOP/Ni ratios of (**a**) 0; (**b**) 0.1; (**c**) 0.3; (**d**) 0.5; and (**e**) 0.8.

**Figure 7:**
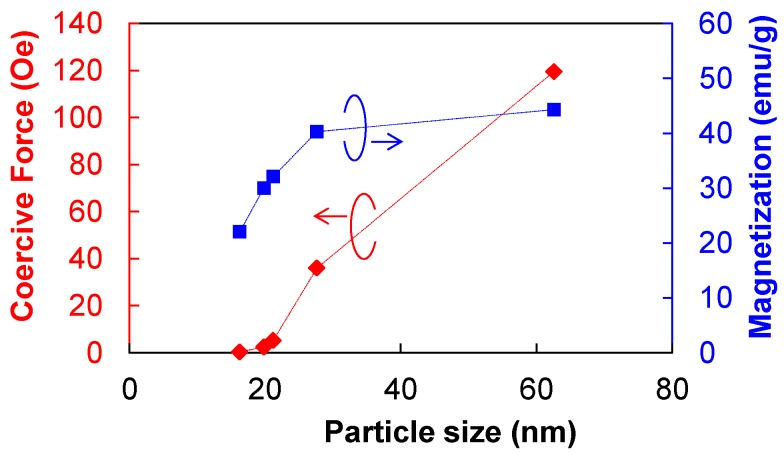
Dependence of coercive forces and saturation magnetization values on the size of the Ni nanoparticles.

Figure 6 shows the magnetization curves of the Ni nanoparticles. The saturation values for magnetization were estimated from the entire graphs, whereas the coercive forces were estimated from the external magnetic fields at the magnetizations of zero in the enlarged graphs. The relationships between the particle size and saturation magnetization, and coercive force are summarized in Figure 7. The coercive force decreases with increase in the TOP/Ni ratio and is almost zero at the TOP/Ni ratio of 0.8, indicating that the Ni nanoparticles become superparamagnetic when the particle size is below 20 nm. Similarly, the saturation magnetization value also decreases as the particles become smaller. When the TOP/Ni ratio is higher than 0.3, the magnetization curves show horizontal shifts toward negative direction as shown in the enlarged graphs. This shift can be caused by the exchange bias effect due to the magnetic exchange interaction between ferromagnetic and antiferromagnetic phases. It was reported that Ni(core)@NiO(shell) nanoparticles indicated the exchange bias effect because Ni is ferromagnetic and NiO is antiferromagnetic and it became more pronounced as the particle size decreased [32,33]. The present result also indicates that the horizontal shift becomes more pronounced as the particle size decreases with increase in the TOP/Ni ratio. Although the XRD spectra of the obtained Ni nanoparticles do not exhibit peaks corresponding to NiO, thin NiO layers can be formed naturally on Ni surfaces in an air atmosphere and may have incomplete crystal structures or disorder which are not easy to be identified by XRD [32,33]. In the Discussion section, the surface analysis of the Ni nanoparticles is presented.

## 3. Discussion

The above results reveal that the size of the Ni nanoparticles can be reduced as an addition of TOP is increased so that the coercive force becomes 0 when the size is below 20 nm at the TOP/Ni ratio of 0.8. On the other hand, the saturation magnetization value gradually decreases as the size is reduced, but the reason is not clear. In this part, the reason about the magnetization decrease will be clarified from the composition and surface analyses of the Ni nanoparticles.

**Figure 8:**
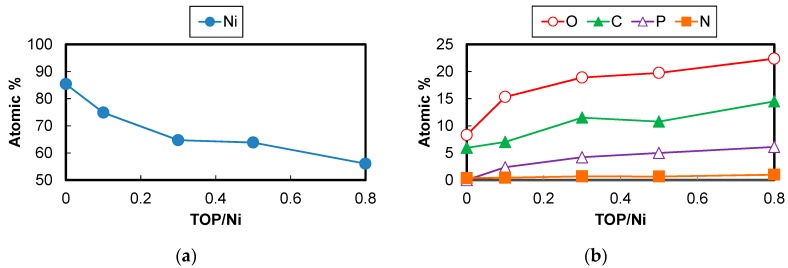
(**a**) Ni atomic percentage and (**b**) O, C, P, and N atomic percentages for the Ni nanoparticles at the TOP/Ni ratios from 0–0.8.

**Figure 9:**
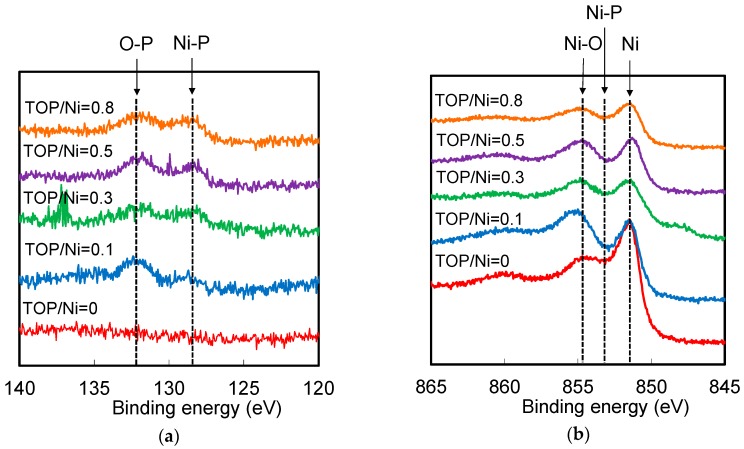
X-ray photoelectron spectroscopy (XPS) (**a**) P 2p3/2 and (**b**) Ni 2p3/2 spectra of the Ni nanoparticles at the TOP/Ni ratios from 0–0.8.

**Figure 10:**
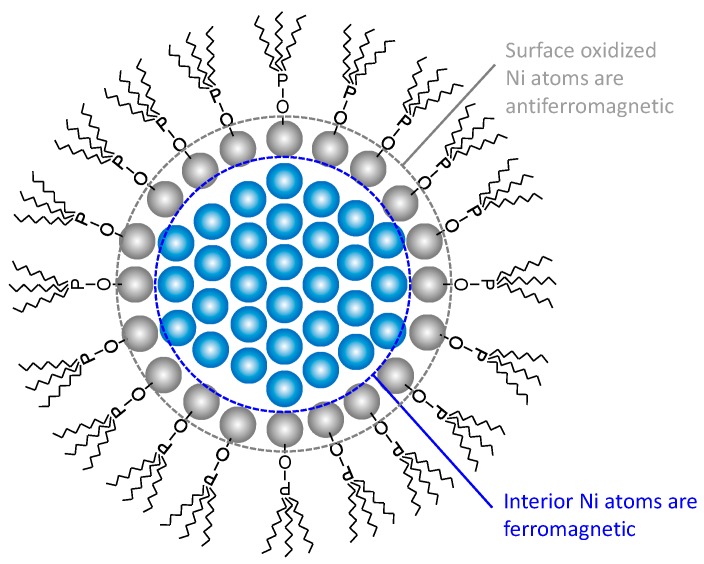
The supposed model of the Ni nanoparticles capped by trioctylphosphine oxides (TOPO).

The atomic composition of the Ni nanoparticles was measured and the results are shown in Figure 8. The Ni atomic percentage decreases, while the O, C, P, and N atomic percentages increase with an increase in the TOP/Ni ratio. Since the particles become smaller with increase in the amount of TOP, the amount of capping layers is thought to increase with increase in the amount of TOP, as a result of which the Ni atomic percentage is reduced. The N atomic percentage is much lower than the P atomic percentage, indicating that the capping layer is mainly derived from TOP and not from OA. The O, C, and P atomic percentages increase with increase in the TOP/Ni ratio. The O and C atomic percentages are higher than the P atomic ratio. The O atomic percentage is already high even in the absence of TOP. Since the N atomic percentage is very small, the O is considered to be derived from the oxidation of the Ni nanoparticles and not from OA. Previous studies have also indicated that Ni surfaces continue to be sensitive to oxidation even after they are covered with capping layers [22]. As the TOP/Ni ratio increases, the O atomic percentage increases gradually in response to the increase in the P atomic percentage. Since TOP is also sensitive to oxidation and the Ni surface can act as a catalyst for oxidation [25], the increase in the O atomic percentage may be due to the oxidation of TOP. Trioctylphosphine oxides (TOPO) may also deposit on the Ni surface as capping layers to form Ni nanoparticles [22,23,24]. In order to ascertain whether TOP is oxidized on the Ni surface, X-ray photoelectron spectroscopy (XPS) P 2p3/2 and Ni 2p3/2 spectra were acquired for the Ni nanoparticles and the results are shown in Figure 9. All the Ni nanoparticles synthesized with TOP exhibit a peak corresponding to the O-P bond in the XPS P 2p3/2 spectra. This result also proves that the TOP present on the Ni nanoparticles is oxidized. A peak corresponding to the Ni-O bond is shown in the XPS Ni 2p3/2 spectra of the Ni nanoparticles synthesized with and without TOP. It is considered that the surface of Ni nanoparticles are oxidized and connected with TOPO by Ni-O bond. Since peaks corresponding to NiO are not shown in the XRD spectra of the obtained Ni nanoparticles, the oxidized surface layers might be very thin or not crystalline. The supposed model of the Ni nanoparticles capped by TOPO is shown in Figure 10. Additionally, since the interior Ni atoms are ferromagnetic and surface-oxidized Ni atoms are antiferromagnetic, the exchange bias effect occurs in the magnetization curves [32,33].

Since the surfaces of the Ni nanoparticles are oxidized, their surfaces no longer have magnetic properties, resulting in a decline in the saturation magnetization with decrease in the particle size. The surface atomic ratios of the Ni nanoparticles can be calculated from the mean diameters as shown in Figure 5. The Ni atomic percentages are shown in Figure 8. The saturation magnetizations of the Ni nanoparticles without the capping layers (*M_S_calc_*) were calculated based on the assumption that the Ni nanoparticles are simple spherical and have fcc structures, bulk Ni has a saturation magnetization value of 54 emu/g (*M_S_Ni_*) as the physical value, and the interior Ni atoms are ferromagnetic, whereas the outermost surface Ni atoms are antiferromagnetic. Although the thickness of oxidized layers is not clear, it is assumed to be very thin because of no peaks for Ni oxide in the XRD spectra and the simple model that only outermost surface Ni atoms are oxidized is adapted:

(1) $$V=\frac{\pi D_{m}^{3}}{6}$$

(2) $$A=\pi D_{m}^{2}$$

(3) $$N_{V}=4/a^{3}$$

(4) $$N_{A}=2/\frac{\sqrt{3}}{2}a^{2}$$

(5) $$M_{S\_calc}=M_{S\_Ni}\times R_{Ni}\times \frac{VN_{V}-AN_{A}}{VN_{V}}$$

In the above equations, *D_m_* is the mean diameter of the Ni nanoparticles, *V* is the volume of the Ni nanoparticles, *A* is the surface area of the particles, *N_V_* is the number of the Ni atoms per unit volume, *N_A_* is the number of the Ni atoms per unit surface area, a is the lattice constant of Ni (0.352 nm) and *R_Ni_* is the Ni atomic ratio of the Ni nanoparticles. The equation for *N_V_* is dividing the number of the Ni atoms in the unit cell of fcc structures and the equation for *N_A_* is dividing the number of the Ni atoms on the {111} triangle plane (the closed-packed plane). The calculation was carried out considering the error bars of the mean diameters as shown in Figure 5.

**Figure 11:**
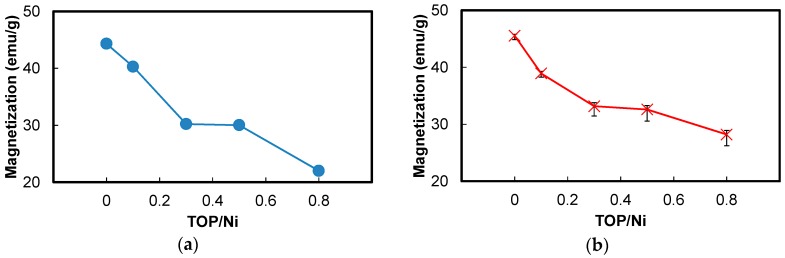
Comparison between the (**a**) measured and (**b**) calculated saturation magnetization values of the Ni nanoparticles at the TOP/Ni ratios from 0–0.8. The calculations were conducted based on the assumption that the interior atoms in the Ni nanoparticles are ferromagnetic, whereas the outermost surface atoms are antiferromagnetic. The error bars in the calculation result was estimated considering the error bars of the mean diameters.

Figure 11 compares the measured and calculated saturation magnetizations for Ni nanoparticles synthesized at the TOP/Ni ratios in the range of 0 to 0.8. Although there is a slight difference between the measured and calculated values, the saturation magnetization values decrease approximately in similar fashion as the TOP/Ni ratio is increased. Therefore, the decrease in the saturation magnetization is mainly attributed to increase in the amount of capping layers and the number of oxidized surface atoms with the decrease in the particle size. Further, the differences between the measured and calculated values increase with increase in the TOP/Ni ratio which means decrease of the particle size. Since the exchange bias effect becomes pronounced as the particle size decreases, the effect of surface layers can be enhanced by decrease of the particle size so that the measured magnetization is lowered much more. We think that this behavior may be also attributed to the presence of Ni phosphides, which could also result in a decrease in the magnetic properties. The formation of Ni_2_P derived from Ni-phosphine complexes at over 300 °C has been reported previously [34,35]. Since our reaction temperature is much lower than 300 °C, a small amount of Ni phosphides is expected to form if the same reaction were to occur. The XPS spectra shown in Figure 9 exhibit a peak corresponding to the Ni-P bond.

The above results prove that the Ni nanoparticles decrease in size with increase in the TOP/Ni ratio and exhibit superparamagnetism at a particle size lower than 20 nm. Further, hysteresis loss can be decreased by reducing the particle size. On the other hand, the saturation magnetization values decrease upon reduction in the particle size because the ratios between the amount of capping layers and amount of oxidized surface atoms increase. In the next study, we will be examining various methods to enhance the saturation magnetization, such as by the decomposition of capping layers.

## 4. Materials and Methods

### 4.1. Materials

Ni(II) acetylacetonate (Ni(acac)_2_, 95%) and oleylamine (OA, 70%) were purchased from Sigma-Aldrich (St. Louis, MO, USA). Trioctylphosphine (TOP, 96%) and acetone (99%) were purchased from Wako Pure Chemical Industries (Osaka, Osaka, Japan). The chemicals were not subjected to further purification and were used as-received.

### 4.2. Synthesis of Ni Nanoparticles

Ni nanoparticles were synthesized by a one-pot process involving the reduction of Ni(acac)_2_ in OA with various amounts of TOP. Ni(acac)_2_ (60 mmol), OA (480 mmol), and TOP (0–48 mmol) were mixed in a flask and subjected to a N_2_ atmosphere at room temperature after evacuating the flask by a vacuum pump . The molar ratio of TOP to Ni(acac)_2_ ranged from 0–0.8. The solution mixture was heated to 130 °C and stirred for 30 min to dissolve the reagents completely under a N_2_ flow of 1 L/min. Following this, the solution mixture was stirred for a further 60 min at 220 °C and cooled to room temperature using ice water. The black precipitate obtained was rinsed with excess acetone and centrifuged for 20 min at 3000 rpm. This procedure was carried out at least twice to remove any residual impurities. The final particles were obtained after drying the precipitate under vacuum at room temperature.

### 4.3. Characterization

X-ray diffraction (XRD) spectra were collected to identify the crystal structures of the particles using an RINT-TTR (Rigaku, Akishima, Tokyo, Japan) instrument with a Cu-Kα X-ray source at a voltage and current of 50 kV and 300 mA, respectively. The crystallite size of the particles was estimated by Scherrer’s equation. Transmission electron microscopy (TEM) (observations were carried out for analyzing the morphology of the particles. A JEM-2000EX instrument (JEOL) (JEOL, Akishima, Tokyo, Japan) was used for this analysis with an acceleration voltage of 200 kV. The samples were prepared by dropping a hexane dispersion of the particles onto Cu microgrids having an amorphous carbon film and drying at room temperature. The mean particle diameters and standard deviations were estimated by measuring the sizes of over 200 particles in the TEM micrographs. When the particle shape was irregular form, the diameter was determined by averaging long and short diameters. The equations used for calculating the mean diameter (*D_m_*) and standard deviation (*SD*) are provided below:

(6) $$f\left(D\right)=\frac{n}{N}$$

(7) $$D_{m}=\sum D\times f\left(D\right)$$

(8) $$SD=\sqrt{\sum {\left(D-D_{m}\right)}^{2}\times f\left(D\right)}$$

In the above equations, *D* is the diameter of the particles, *n* is the number of particles with diameter *D*, *N* is the total number of particles measured, and *f*(*D*) is the frequency of particles with diameter *D*.

Magnetization curves of the particles were measured by vibrating sample magnetometry (VSM) using a TM-VSM311483-HGC (Tamakawa, Sendai, Miyagi, Japan) instrument. Magnetic fields up to 20 kOe were applied. Inductively-coupled plasma (ICP) analysis was carried out to determine the compositions of Ni and P using CIROS-120EOP (Rigaku, Akishima, Tokyo, Japan). The infrared absorption method by combustion with EMIA-920V (Horiba, Kyoto, Kyoto, Japan) was used to determine the weight composition of C. On the other hand, the infrared absorption method by inert gas fusion with EMGA-820 (Horiba, Kyoto, Kyoto, Japan) was used to determine the weight composition of O. The thermal conductivity detection method by inert gas fusion was used to determine the weight composition of N using EMGA-820 (Horiba, Kyoto, Kyoto, Japan). The atomic percentages of Ni, O, C, P and N were estimated from dividing the weight compositions by the atomic weight and the total molar quantity. X-ray photoelectron spectroscopy (XPS) analysis was conducted using an Al-Kα X-ray source to determine the chemical state of the particles.

## 5. Conclusions 

Ni nanoparticles were synthesized at 220 °C by the reduction of Ni(acac)_2_ in OA with TOP as the capping layer. Complete reduction of Ni(acac)_2_ occurred when the reaction duration was over 30 min. Further, the size of the Ni nanoparticles decreased as the TOP/Ni ratio increased. When the TOP/Ni ratio was 0.8, the Ni nanoparticle size was less than 20 nm and coercive force was almost 0, owing to superparamagnetism. On the other hand, the saturation magnetization also decreased with reduction in the Ni nanoparticle size. The P and O atomic ratios increased in the same manner with increase in the TOP/Ni ratio, indicating that the main capping layers were composed of TOPO, derived from the oxidation of TOP. The formation of TOPO was also confirmed by the XPS spectral results. The Ni atomic percentage decreased as the Ni nanoparticles reduced in size. The saturation magnetization values of the Ni nanoparticles without capping layers were calculated based on the assumption that the atoms within the nanoparticles are magnetic, whereas the surface oxidized atoms are not magnetic. The measured and calculated saturation magnetization values decreased in approximately similar fashion as the TOP/Ni ratio increased, indicating that the decrease in the saturation magnetization can be mainly attributed to the increase in the amount of capping layers and oxidized surface atoms. This study indicate that a hysteresis loss can be decreased by adding TOP into Ni nanoparticles to reduce the size, which is suitable for passive components used under high frequencies, but the saturation magnetization is necessary to be enhanced on the hand.
